# Two Distinct Genomic Lineages of Sinaivirus Detected in Guyanese Africanized Honey Bees

**DOI:** 10.1128/mra.00512-22

**Published:** 2022-07-18

**Authors:** Declan C. Schroeder, Jaclyn L. Stone, Amy Weeks, Makeba Jacobs, Jeol DaSilva, Shimar Butts, Lorenzo Richards, Shevon Layne, Erwin Miller, Dwight Walrond, Dane Hartley, Dexter Lyken

**Affiliations:** a Department of Veterinary Population Medicine, University of Minnesota, St. Paul, Minnesota, USA; b My Favorite Honey Farm, West Monroe, Louisiana, USA; c Guyana Livestock Development Authority, East Coast Demerara, Lusignan, Guyana; d School of Biological Sciences, University of Reading, Whiteknights, Reading, United Kingdom; Portland State University

## Abstract

Over the past decade or so, PCR-based screening programs have reported that Africanized honey bees (AHB) are also hosts to viruses commonly found in European honey bees. Very little is known about the genomic variants found in AHB. Here, we present two distinct lineages of sinaiviruses in AHB.

## ANNOUNCEMENT

**FIG 1:**
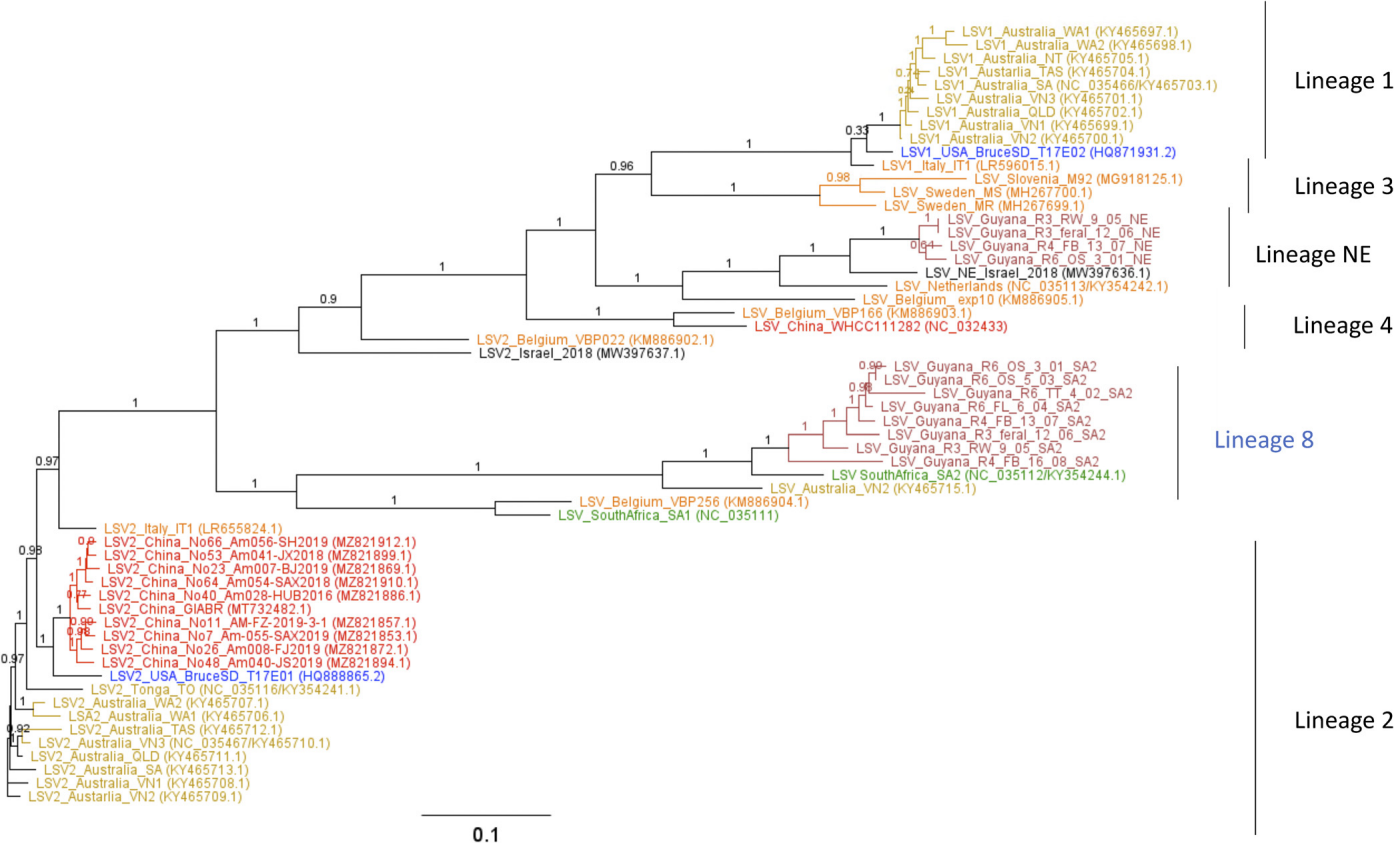
Phylogenetic inference tree, created using FastTree 2.1.11 in Geneious Prime 2021.1.1, showing the location of the Guyanese LSV genome sequences (maroon) relative to Oceania (gold), North America (blue), East Asia (red), Middle East (black), Africa (green), and Europe (orange). Bar represents 1 substitution per 10 nucleic acids. The nodes indicate bootstrap values.

Lake Sinai virus (LSV), family *Sinhaliviridae* within the order *Nodamuvirales*, was first discovered in European honey bee (EHB) colonies in the United States (1). Since then, LSV variants have been detected in multiple bee species across the world (2, 3). LSV has also been detected in African honey bees (4), and the only African LSV genomes published to date are from those found in honey bees collected in South Africa (5). Here, we sequenced and identified two distinct lineages of LSV (Fig. 1) in Guyanese Africanized honey bees (AHB).

Samples made up of 30 whole worker AHB per apiary (Table 1) were pooled and liquefied using the gentleMACS dissociator (RNA 02.01 program; Miltenyi Biotec). The samples represented 3 regions across Guyana, namely, regions 6, 4, and 3. Total RNA was extracted per the manufacturer’s instructions using the NucleoSpin virus RNA-DNA isolation kit (TaKaRa Bio USA). Sequencing was carried out as previously described (6), except that cDNA synthesis was carried out using the template switching (TS) RT enzyme mix (New England Biolabs) with an N6 TS modified random primer (Thermo Fisher Scientific). RNA and DNA quantifications were carried out using the Qubit 4 fluorometer (Thermo Fisher Scientific). Region- and apiary-specific Oxford Nanopore Technologies (ONT) libraries (8 in total; Table 1) were prepared using the rapid barcoding SQK-RBK004 sequencing kit (Oxford Nanopore Technologies). All libraries were sequenced in a single sequencing run using the high-accuracy base-calling model with a minimum Q score of 7 set on an ONT MinION device using one FLO-MIN106 R9 flow cell.

**TABLE 1:** Origin of samples, genomes, and sequence metadata returned after using default Minimap 2.17 settings in Geneious Prime 2021.1.1

Isolate	Top Epi2Me match (GenBank accession no.)	Sample description (30 pooled adult bees)	Region	Community	Sample no.	Barcode	No. of mapped reads	Total no. of reads	Min length (bp)	Max length (bp)	No. of bases	Genome length (bp)	Coverage (×)	SRA accession no.	GenBank accession no.
LSV_Guyana_R6_OS_3_01_NE	NE (NC_035113.1)	5 bees each from 6 hives	6	Sandvoort	3	1	533	11,078	199	3,209	405,408	5,533	73.27	SRX14642749	ON108632.1
LSV_Guyana_R6_OS_3_01_SA2	SA2 (NC_035112.1)						647	11,078	204	3,234	496,029	5,737	86.46	SRX14642750	ON108628.1
LSV_Guyana_R6_TT_4_02_SA2	SA2 (NC_035112.1)	15 bees each from 2 hives			4	2	115	14,165	216	1,881	60,752	5,570	10.91	SRX14642753	ON108637.1
LSV_Guyana_R6_OS_5_03_SA2	SA2 (NC_035112.1)	5 bees each from 6 hives			5	3	1,248	18,069	190	3,149	994,517	5,737	173.35	SRX14642754	ON108633.1
LSV_Guyana_R6_FL_6_04_SA2	SA2 (NC_035112.1)	6 bees each from 5 hives			6	4	463	13,032	187	2,555	371,963	5,737	64.84	SRX14642755	ON108634.1
LSV_Guyana_R3_RW_9_05_NE	NE (NC_035113.1)	5 bees from each of 6 hives	3	La Grange	9	5	557	12,021	181	3,388	437,766	5,596	78.23	SRX14642756	ON108631.1
LSV_Guyana_R3_RW_9_05_SA2	SA2 (NC_035112.1)						452	12,021	202	3,367	358,157	5,737	62.43	SRX14642757	ON108638.1
LSV_Guyana_R3_feral_12_06_SA2	SA2 (NC_035112.1)	Single feral colony		Harmonie	12	6	200	14,830	235	2,455	153,924	5,665	27.17	SRX14642758	ON108636.1
LSV_Guyana_R3_feral_12_06_NE	NE (NC_035113.1)						951	14,830	193	2,871	790,095	5,551	142.33	SRX14642759	ON108630.1
LSV_Guyana_R4_FB_13_07_SA2	SA2 (NC_035112.1)	5 bees each from 6 hives	4	Beehive	13	7	274	21,720	182	2,802	199,407	5,737	34.76	SRX14642760	ON108635.1
LSV_Guyana_R4_FB_13_07_NE	NE (NC_035113.1)						1,066	21,720	168	3,102	844,404	5,783	146.01	SRX14642751	ON108629.1
LSV_Guyana_R4_FB_16_08_SA2	SA2 (NC_035112.1)	7 bees each from 2 hives and 8 each from another 2 hives		Land of Caanan	16	8	232	12,360	183	2,128	198,258	5,740	34.54	SRX14642752	ON108639.1

Consensus genome assemblies and phylogenies were created using default parameters for all software. The reads obtained were reference assembled against the top Epi2Me WIMP hit for each genome (Oxford Nanopore Technologies), which was either the genome submitted under GenBank accession number NC_035112.1 or NC_035113.1, using Minimap 2.17 in Geneious Prime 2021.1.1 and manually curated to correct ambiguities where possible. For each Guyanese LSV consensus genome in lineages NE and 8, reads ranging from 115 to 1,248 bases (Table 1) were used to assemble the near-complete consensus genomes of LSV-8- and LSV-NE-like variants (Fig. 1). All Guyanese LSV genomes were assigned a label to indicate the region of origin, beekeeper code, library, barcode, and finally, the best Epi2Me LSV genome match (Table 1; Fig. 1). The coding regions (5′ and 3′ regions removed) of the new consensus genomes were aligned to available LSV genomes found in NCBI (downloaded March 2022) using Muscle 3.8.425 (7) and visualized using FastTree 2.1.11 (8) in Geneious Prime 2021.1.1.

LSV variants can be subdivided into lineages based on the RNA-dependent RNA polymerase (RdRp) gene and their whole-genome sequences (9, 10). Phylogenetic analysis revealed that two distinct LSV lineages exist in Guyanese AHB (Fig. 1). One clade of Guyanese LSV variants clustered with a variant in lineage 8, named LSV-SA2 (Fig. 1). The second clade of Guyanese LSV variants clustered with variants previously assigned to LSV lineage 1 (10). Here, we show how LSV lineage 1 can be split into two lineages, creating a new lineage, namely, lineage NE (Fig. 1).

### Data availability.

The genome sequences for this project have been deposited at GenBank under the following accession numbers: ON108628 to ON108639. The Oxford Nanopore Technology reads are available under BioProject accession number PRJNA820891. Links to the read files in the SRA for all the new LSV genomes can be found in Table 1.
